# The impact of differing frames on early stages of intersectoral collaboration: the case of the First 1000 Days Initiative in the Western Cape Province

**DOI:** 10.1186/s12961-019-0508-0

**Published:** 2020-01-09

**Authors:** Ida Okeyo, Uta Lehmann, Helen Schneider

**Affiliations:** 1 School of Public Health, University of the Western Cape, Robert Sobukwe Road, Bellville, 7535 Republic of South Africa; 2 School of Public Health and UWC/SAMRC Health Services to Systems Research Unit, University of the Western Cape, Robert Sobukwe Road, Bellville, 7535 Republic of South Africa

**Keywords:** Western Cape Province, South Africa, Intersectoral collaboration, Frames, Policy ideas, Policy formulation, First 1000 Days, Common goals

## Abstract

**Background:**

While intersectoral collaboration is considered valuable and important for achieving health outcomes, there are few examples of successes. The literature on intersectoral collaboration suggests that success relies on a shared understanding of what can be achieved collectively and whether stakeholders can agree on mutual goals or acceptable trade-offs. When health systems are faced with negotiating intersectoral responses to complex issues, achieving consensus across sectors can be a challenging and uncertain process. Stakeholders may present divergent framings of the problem based on their disciplinary background, interests and institutional mandates. This raises an important question about how different frames of problems and solutions affect the potential to work across sectors during the initiating phases of the policy process.

**Methods:**

In this paper, this question was addressed through an analysis of the case of the First 1000 Days (FTD) Initiative, an intersectoral approach targeting early childhood in the Western Cape Province of South Africa. We conducted a documentary analysis of 34 policy and other documents on FTD (spanning global, national and subnational spheres) using Schmidt’s conceptualisation of policy ideas in order to elicit framings of the policy problem and solutions.

**Results:**

We identified three main frames, associated with different sectoral positionings — a biomedical frame, a nurturing care frame and a socioeconomic frame. Anchored in these different frames, ideas of the problem (definition) and appropriate policy solutions engaged with FTD and the task of intersectoral collaboration at different levels, with a variety of (sometimes cross) purposes.

**Conclusions:**

The paper concludes on the importance of principled engagement processes at the beginning of collaborative processes to ensure that different framings are revealed, reflected upon and negotiated in order to arrive at a joint determination of common goals.

## Key messages

1. Limited attention to the role of framing during early decision-making processes for intersectoral collaboration.
2. Different frames of policy problems and solutions reflect different positions on intersectoral collaboration, which can hinder achieving a shared understanding necessary for intersectoral action for child health.
3. Principled engagement process at early stages of intersectoral processes to ensure that frames are surfaced and negotiated as a means of achieving common goals.

## Background

It is increasingly recognised that achieving effective health outcomes requires approaches that extend beyond the provision of health services. As a result, there has been a call for the health sector to work across sectors to effectively address health challenges, a concept referred to as intersectoral collaboration [1–3]. Intersectoral collaboration for health has been defined as “*a recognised relationship between part or parts of the health sector with parts of another sector which has been formed to take action on an issue to achieve health outcomes (or intermediate health outcomes) in a way that is more effective, efficient or sustainable than could be achieved by the health sector acting alone*” [4].

The First 1000 Days (FTD) began as a global advocacy concept to draw attention to the impact of nutrition on long-term health and development [5]. The FTD window, from conception to 2 years of age, represents a period of vulnerability due to the rapid development processes that occur, and which is particularly sensitive to early life adversity associated with poverty, poor nutrition and substance abuse. There is an increasing recognition of the impact of early life determinants (adequate nutrition, stimulation and responsive caregiving) on a child’s health and development throughout the lifespan. Evidence shows that intervening in this period has major benefits in improving health outcomes and reducing inequalities [6]. The FTD has thus been advocated globally as a target area for interventions focusing on nutrition, early childhood development and mental well-being, especially for developing countries, where 39% of children younger than 5 years are documented as being at risk of not reaching their developmental potential [7].

Although the FTD initiative is not a national policy in South Africa, the period has been recognised in key policy frameworks such as the National Development Plan [8] and the National Integrated Early Childhood Development Policy [9], which highlight action in early childhood as crucial in ensuring national development and growth. The Western Cape Province, on the other hand, has recognised the significance of the FTD in ensuring wellness and enabling children to thrive and reach their full potential. Although noted to be performing better than other Provinces in South Africa, 37% of children live in poor households (households earning a monthly income below US$81.01) and 11% live in households where hunger is reported, making them vulnerable to poor developmental outcomes [10]. In addition, the Province has the highest rates of drug-related crime in the country [11] and high levels of alcohol and substance abuse have been identified as the main contributing factors to domestic violence and child abuse [12].

As a response to the growing number of at-risk children and major social challenges, such as high levels of violence, the province launched the FTD Initiative in 2015 under its strategic goal to “*increase wellness and safety and tackle social ills*” [13]. Based on recommended concepts of nurturing care [14] adapted for the Western Cape context, the goal of the FTD initiative is to improve outcomes for children in terms of nutrition, health (including maternal health), education (early learning), support and parenting, and protection and safety. The mandate to implement the FTD initiative was assigned to the Health Programmes Directorate of the health sector and a FTD executive committee consisting of health actors was formed to take the initiative forward.

The health sector is favourably situated as a lead sector and entry point for organising an intersectoral response to social determinants of health during the FTD. This is because the health sector frequently interacts with women, children and their families from conception to early childhood, creating the opportunities to address psychosocial factors during routine visits and link caregivers with other available supportive services [15, 16]. Despite consensus on the central roles the health sector can play, there is less agreement on how the health sector is to mobilise other sectors or the appropriate course of action to follow within the FTD; this includes the roles and responsibilities of sectors responsible for education and social services.

Although advocated as a key strategy for early childhood development, tackling the FTD is bound to share the challenges of achieving intersectoral collaboration documented for other health issues. Collaboration is often constrained by the vertical organisation of sectors, which makes it difficult to co-ordinate, ensure horizontal accountability for service delivery, and measure effectiveness and impact as well as by the time and effort required in establishing cross-sectoral relationships [3, 17–20].

An additional challenge presents during early decision-making stages where stakeholders have to agree on common goals and the way forward, which may be particularly difficult where the boundaries of the problem and its solutions are unclear and uncertain [21]. Differing perspectives and backgrounds can create contestation, and if there is no shared understanding of what partners can achieve together, intersectoral collaboration may never get out of the starting blocks. The lack of consensus on problem definitions related to early childhood development (ECD) impacts the ability to advance global priority for ECD [22]. Similarly, efforts to address undernutrition and ECD reveal the impact of unclear policy solutions on sustaining commitment to action and developing concrete implementation plans [23, 24].

The process of translating evidence on intersectoral collaboration into effective policy therefore continues to challenge health systems and policy-makers. There is a scarcity of evidence on intersectoral action in low- and middle-income contexts as well as on policy and public administrative processes [3, 15, 18]. Therefore, as part of a broader study of intersectoral collaboration during policy formulation and implementation, this paper explores how meanings of a policy problem are constructed, especially in highly contested policy issues with differing opinions on solutions at early stages of policy development. In particular, based on an analysis of policy and policy-related documents on FTD, we focus on the role that frames (of both problems and solutions) play during early decision-making stages of intersectoral collaboration and the possible implications for advancing to policy formulation and implementation. The study highlights pertinent challenges surrounding early stages of intersectoral collaboration that policy-makers in similar contexts should consider in order to approach collaboration in ways that are more likely to lead to sustained success.

### Role of ideas and frames in policy processes

Ideas are products of our own cognition that influence how we interpret our surroundings and construct the social world, shaping worldviews, casual beliefs, frames, societal norms and cultures [25–29]. Frames are a package of ideas that act as ‘cognitive maps’ or channels through which meaning is structured and preferences expressed, and which serve as reference points for viewing new information [30, 31]. In the development of policies, frames serve to focus attention on a selected part of the problem and specific solution while simultaneously diverting attention from any other solution that may be present [29, 32]. During policy formulation, frames evolve as actors interact in defining, debating and challenging problem definitions and solutions, which may become integrated into existing frames or can evolve into new definitions of the problem and explanation for the policy issues [30].

Various forms of framing analysis are available to those interested in studying frames [31, 33–35]. In this study, we were interested in how different viewpoints and interests were articulated in policy documents as well as the arguments used to support them. We applied Schmidt’s typology of ideas [27] as it offered a way to organise data in order to elicit frames from examining policy ideas, including how they are conveyed through the discourse in policy documents.

Schmidt conceptualises ideas underpinning discourses and frames at three levels of generality [27]. The first level refers to specific policy ideas or policy solutions to identified problems. The second level describes general policy programmes that define the problem, goals to be achieved, methods to be applied and the objectives. These programmes reflect the underlying assumptions orienting policy and can be thought of as programmatic beliefs that operate between worldviews and specific policy ideas. Ideas as policy programmes (programmatic ideas) are usually found in the centre of most policy debates and are favoured by policy actors as they help actors determine solutions to policy problems [36]. The third level considers a more general level of ideas, which includes public philosophies or worldviews that frame the policy within a deeper set of ideas, values and principles of knowledge that reflect larger constructions of society, economics or politics. While ideas in the first and second level are often discussed and debated, philosophies that underpin policies and programmes are normally in the background [27, 28].

## Methods

A qualitative documentary analysis [37, 38] of ideas and underlying frames was adopted for this study. The analysis of documentary sources is recognised as a valuable qualitative analysis method and has been used, amongst others, to examine policy responses to the social determinants of health [37–40]. Although examining written text may not reveal negotiations and contestation during policy-making, policy documents illustrate the outcomes of a policy process and can provide insights into underlying values, ideas or meanings that influence policy action.

The document analysis process sought to answer the empirical question of how policy ideas regarding the FTD reflect overall structures of meaning in frames. The document selection process was conducted by the main author, as part of her doctoral studies, who specifically looked for documents focused on the FTD period and not on ECD as a whole, which stretches from 0 to 9 years, during which other government sectors (notably education) may have more prominent roles than the health sector. However, some of the FTD-relevant texts were embedded in or had to be inferred from ECD-related policies, especially those released before 2014, when the FTD concept was not as yet widely in circulation.

The document selection process was iterative and was done over a period of 8 months (February to October 2018), as new initiatives linked to the FTD were unfolding at national and provincial level. Key informant interviews conducted during the course of document selection ensured that all the relevant documents shaping the initiative were included. Document selection occurred in three stages. The first stage was informed by the researchers’ observations of key provincial events related to the FTD and attendance at two different intersectoral working group meetings. Documents received through interactions with stakeholders were largely health sector strategies, policy reports, newsletters and global literature that key informants felt had shaped the initiative provincially. In a second stage, references from the documents received in the first stage were followed and searched for in the Western Cape Provincial website. Annual provincial reports and performance plans across sectors were scanned to identify whether there was any text referring to the FTD, with a deliberate effort to explore if the FTD was prioritised in policies of Departments of Education, Social Development, and Community and Safety. In the last stage, broader national level policies or documents that focused on the FTD were identified, including maternal and child health policies and strategies as well as relevant international and national scientific literature. Through this process, a total of 34 documents were obtained and analysed (listed in Table 1).

**Table 1:** Documents analysed

Document origin/authors	Type of document	Year	Document title	Frames
Global documents or strategies (largely used to shape the initiative locally)				
1. World Health Organization	Strategy	2016	Global Strategy for Women’s, Children’s and Adolescents’ Health (2016–2030)	Biomedical, socioeconomic, nurturing care
2. Black et al.	*Lancet* series	2016	Early Childhood Development Coming of Age: Science Through the Life Course	Nurturing care, socioeconomic
3. Britto et al.	*Lancet* series	2016	Nurturing Care: Promoting Early Childhood Development	Nurturing care
4. Richter et al.	*Lancet* series	2017	Investing in the Foundation of Sustainable Development: Pathways to Scale Up for Early Childhood Development	Socioeconomic
5. World Health Organization	Framework	2018	Nurturing Care for Early Childhood Development: A Framework for Helping Children Survive and Thrive to Transform Health and Human Potential	Nurturing care, socioeconomic
6. Harvard University	Report	2010	The Foundations of Lifelong Health are Built in Early Childhood	Socioeconomic
7. United Nations	General Assembly Resolution	2015	Transforming Our World: The 2030 Agenda for Sustainable Development	Socioeconomic
National documents Whole of society policies (that were FTD-sensitive)				
8. Department of Social Development	Policy	2015	National Integrated Early Childhood Development Policy	Nurturing care
9. National Planning Commission	Strategic plan	2011	National Development Plan Vision 2030	Nurturing care
10. UNICEF	Policy/Plan	2005	National Integrated Plan for Early Childhood Development (2005–2010)	Socioeconomic
11. Department of Basic Education	Policy	2015	The South African National Curriculum Framework for Children from Birth to Four	Nurturing care
Health sector-specific policies				
12. National Department of Health	Policy	2012	Strategic Plan for Maternal, Newborn, Child and Women’s Health and Nutrition in South Africa (2012–2016)	Biomedical
13. National Department of Health	Report	2014	National Report for the Mid-Term Review of the Strategic Plan for Maternal, Newborn, Child and Women’s Health and Nutrition in South Africa (2012–2016)	Biomedical
Non-sector-related documents and journal articles				
14. Morgan, B.	Report	Not dated	Relationships Matter Most, Especially in the First 1000 Days. The Interdisciplinary Neuroscience of Early Childhood Development: A Unifying Narrative	Socioeconomic
15. Children’s Institute, University of Cape Town	Report	2017	South African Child Gauge 2017	Nurturing care, socioeconomic
16. Children’s Institute, University of Cape Town and Ilifa Labantwana	Report	2016	South African Early Childhood Review 2016	Nurturing care, socioeconomic
17. Turner & Honikman	Journal article	2016	Maternal Mental Health and the First 1000 Days	Nurturing care
18. English et al.	Journal article	2017	‘First 1000 Days’ Health Interventions in Low- and Middle-income Countries: Alignment of South African Policies with High-quality Evidence	Biomedical
Provincial/subnational documents Whole-of-society policies or plans (that anchored the FTD)				
19. Western Cape Government	Strategy	2014	Provincial Strategic Plan 2014–2019	Socioeconomic
20. Western Cape Government	Declaration	2011	Cape Town Declaration on Wellness	Socioeconomic
21. Western Cape Government	Strategy	2011	Investing in the Early Years, Integrated Provincial Early Childhood Development Strategy (2011–2016)	Nurturing care
22. Western Cape Department of Health	Strategy	2014	Healthcare 2030: The Road to Wellness	Biomedical, nurturing care
Health-sector				
23. Perinatal Task Team	Report	2016	First 1000 Days Rapid Situational Analysis for the Western Cape Survive, Thrive, Transform	Biomedical
24. Perinatal Task Team	Framework	2016	Intervention Framework to Guide Service Planning for the First 1000 Days	Biomedical
25. FTD executive committee	Report	2016	The First 1000 Days Initiative, Cape Town, South Africa	Socioeconomic
26. Western Cape Department of Health	Newsletter	2016	Western Cape Government Research Newsletter 2016	Biomedical
27. Western Cape Department of Health	Newsletter	2017	Western Cape Government, Research Newsletter 2017	Nurturing care
28. Thanjan	Report	2017	Report on the First Round of the First 1000 Days Roadshows Conducted in the Cape Town Metro between April–September 2016	Nurturing care
29. FTD executive committee	Report	2017	Provincial Strategic Plan Goal 3: Increase Wellness and Safety, Reduce Social Ills. Project Charter 2017/2018. Project: The First 1000 Days (FTD) Initiative	Nurturing care
30. Western Cape Department of Health	Report	2015	Western Cape Government, Department of Health Annual Report 2015/2016	Nurturing care
31. Western Cape Department of Health	Report	2016	Western Cape Government, Department of Health Annual Report 2016/2017	Nurturing care
Social Development				
32. Western Cape Department of Social Development	Report	2018	Western Cape Government, Department of Social Development Annual Report (2018/2019)	Nurturing care
33. Western Cape Department of Social Development	Plan	2018	Western Cape Government Department of Social Development Service Delivery Improvement Plan	Nurturing care
34. Western Cape Department of Social Development	Report	2018	Western Cape Government, Department of Social Development Annual Performance Plan 2017/2018	Nurturing care

Once the selection process was complete, documents were initially read to establish their main content, followed by the coding of each document in Microsoft Excel using a priori coding framework based on the conceptualisation of policy ideas by Schmidt [27]. The document extraction Excel sheet is provided as Additional file 1. An example of the deductive coding process is shown in Table 2, where ideas as solutions were coded as statements that referred to what each document identified as the solution/s to the problem. Ideas as programmes were used to code the ‘how’ of the policy solution/s including instruments or the detailed approach mentioned. Based on the ‘what’ and the ‘how’, the underlying worldviews were coded as to why those particular solutions and programmes were chosen and which arguments were used to support that statement. During the coding process, text speaking to the rationales used to justify the focus on early childhood development or the FTD as well as any statements on intersectoral collaboration were extracted.

**Table 2:**
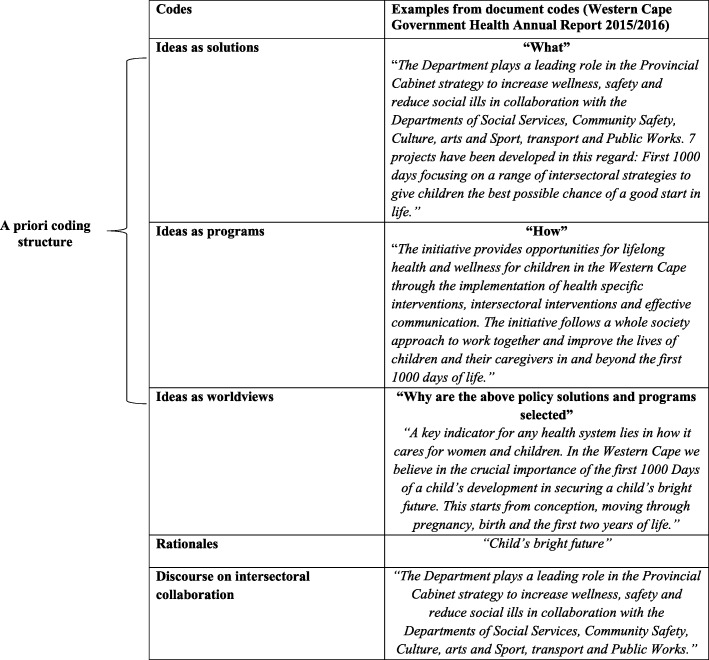
Coding process

Following the thematic analysis approach [37], coded texts were checked and later organised into three broader themes, which we consider frames (Table 3). Although the coding was done by the main author, the analysis process was discussed with the co-authors, after which the naming of the frames and overall structure of the findings was developed. This analysis process began by identifying general patterns through comparing coded segments and grouping policy solution/s and the accompanying arguments that were similar. At this stage problem definitions for each of the solutions were inferred through examining the accompanying arguments and worldviews. This allowed an initial generation of three main problem definitions and matching policy solution/s. Thereafter, frames of the problem definitions and policy solution/s referred to were established by constant comparison with the rest of the data. This led to the identification of three main frames. Afterwards we sought to identify the relationship between frames based on the primary audience that each frame targeted and whether these frames made sense in relation to the entire data set.

**Table 3:** Frames, levels and problem definitions

Levels	Frames	Problem definitions (What is the problem?)	Policy solutions (What is the solution?)
Individual	Biomedical	1. FTD as a maternal and child health mortality problem: “*Safeguarding and preserving the lives of mothers during childbirth, is one of the globally accepted essential functions of a health care system. The maternal mortality ratio is therefore viewed as one of the key markers of the effectiveness of health care systems globally. Western Cape Government Health is therefore committed to reducing maternity mortality, in line with this imperative*.” (First 1000 Days Rapid Situational Analysis Situational Analysis, p. 82, 2016)	“*Improve maternal, perinatal and child mortality by addressing avoidable causes of deaths”* (Intervention Framework in Situational Analysis, p. 85, 2016)
Family	Nurturing care	2. FTD as an early childhood development problem: “*Overwhelming scientific evidence attests to the tremendous importance of the early years for human development and to the need for investing resources to support and promote optimal child development from conception. Lack of opportunities and interventions, or poor quality interventions, during early childhood can significantly disadvantage young children and diminish their potential for success*” (NIECD, p. 8, 2015)	“*Provision of universal developmentally appropriate early learning opportunities for young children from birth …*. *Review and strengthening of a comprehensive national food and nutrition strategy … Support for pregnant women, new mothers/fathers and children under 2 years of age*” (NIECD, p. 64, 2015)
Community/societal	Socioeconomic	3.FTD indicating the need to address social determinants of health: *“The Western Cape Government acknowledges that we have a society that still carries the burdens of inequity....The Western Cape Government is therefore committed to promote wellness in communities in order to ensure safety, health and inclusivity across all communities within the province*.” (Provincial Strategic Plan, p. 36, 2014)	“*A whole-of-society approach to improving people’s lives – an approach built on partnerships with citizens, civil society, business, and other spheres of government*” (HealthCare 2030, p. 65, 2014)

## Results

### The rise of attention to the FTD: global moments and local contexts

**Fig. 1:**
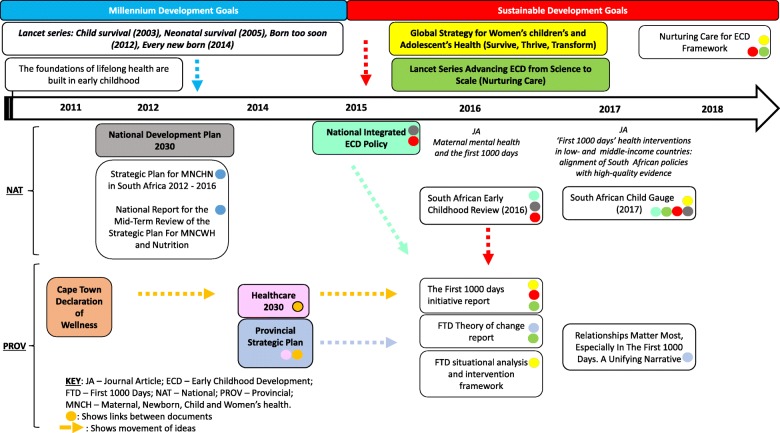
Flow of ideas from global to national contexts and the culmination of ideas at the provincial level

The FTD can be thought of as an idea whose ‘time has come’, a phrase from agenda-setting theories [41] indicating the rise of attention to the FTD at the global, national and subnational levels shown through some of the documents in Fig. 1. Global moments are responsible for creating the original awareness, attention and priority for the FTD. The idea was significantly propelled by a 2008 *Lancet* series on maternal and child undernutrition that made the scientific case for the FTD period being crucial to improving nutrition and development [42]. Additionally, the establishment of the 1000 Days Partnership, a United States-based hub, highlighted the importance of this period and, as a response, international institutions, development organisations and the private sector acted to scale up nutrition interventions [5]. The period of the Sustainable Development Goals (SDGs) ushered a new policy window that allowed sustained attention to the FTD by linking child survival to early child development. Increasing global recognition at the start of the SDG era sought to argue that, while child survival was improving, children were not realising their human potential and contributing to sustainable development.

In contrast to the Millennium Development Goals (MDGs), the SDGs showed a greater appreciation of the interrelatedness of goals and targets and placed an emphasis on collaboration between sectors. This led to increasing interest in potential platforms and opportunities to deliver intersectoral interventions, particularly in the FTD. The sentiments in the SDGs are also reflected in the WHO Global Strategy for Women’s, Children’s and Adolescents’ Health. The Global Strategy stretched the frame of child survival by emphasising aspects of early childhood development. Thereafter, the *Lancet* series on Advancing Early Childhood Development [14] became the point of reference on the severity, causes, costing and solutions to the challenges facing early childhood development, promoting the concept of ‘nurturing care’ as a holistic approach to ensuring child wellbeing.

South African national documents from the health sector predominantly reflect the ideas of child survival, which were advocated during the MDG era through maternal and child health strategies developed in 2012 [43]. Although child survival ideas in national health sector policies have largely remained unchanged, circulation of the global discourse regarding the FTD and the SDGs appeared to occur at an opportune time when the South African Social Development sector released the National Integrated Early Childhood Development policy (NIECD), an ECD policy that provided an overarching multisectoral-enabling framework for ECD services. The NIECD was particularly relevant as it embraced the child development goals of the SDGs and placed a high priority on the FTD period [9].

The NIECD was also crucial as it outlined a comprehensive service package for children that mandated the health sector to be in the forefront of providing services for early childhood through the support of caregivers [9]. Furthermore, this idea of attention to early years seemed favourable nationally as it had been previously emphasised by the National Development Plan [8], which proposed that a focus on addressing the needs of children in the early years would enhance human potential and therefore assist in reducing poverty.

At the subnational level, support for the FTD as an idea emerged as a convergence of the global moments outlined above, the national discussions surrounding the NIECD policy and shifts in thinking at provincial level (Fig. 1). In 2011, the Western Cape Provincial Government issued the Cape Town Declaration of Wellness, which promoted a holistic approach to child wellness in order to address poor nutrition and vulnerabilities due to violence [44]. This was followed by the development of a “*whole of society approach*” expressed in documents such as Healthcare 2030 [45] and the Provincial Strategic Plan [13], which aligned themselves with intersectoral approaches surrounding the FTD such as nurturing care and the Survive, Thrive, Transform framework. This broad mandate led to a series of specific policy initiatives to address the FTD, which positioned the health sector as the lead department in organising the range of intended activities to address the FTD. Other than health sector-specific documents, the FTD also featured in annual reports of the Department of Social Development (Table 1), but not in two other key sectors – Education and Community and Safety.

In summary, an alignment of global discourses and key moments between 2012 and 2018, notably the launch of SDGs, the *Lancet* series on advancing ECD and the WHO Global strategy for Women’s Children’s and Adolescent’s Health, built momentum for FTD. Global ideas surrounding the FTD found fertile ground in South Africa in the National Development Plan and the NIECD as a means to reduce inequality and poverty. Despite national health sector policies remaining set in child survival mandates of the MDG era, a growing interest in whole-of-society approaches in the Western Cape Provincial government, aligned with the global notions of nurturing care and intersectoral action, led to the prioritisation of the FTD provincially.

### Differing frames, problem definitions and policy solutions

**Fig. 2:**
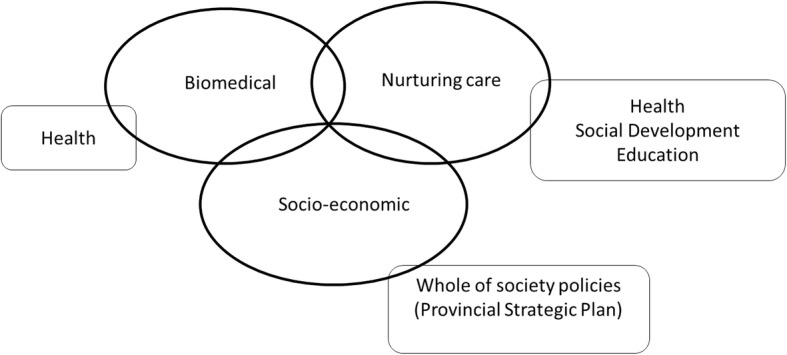
The intersection of frames and sectors within the First 1000 Days Initiative

Although the documents analysed were in agreement on the need to focus on the FTD period, there were a variety of ideas on the ‘what’, ‘how’ and ‘why’ of the FTD. Three broad policy frames on the FTD were identified (Table 3), which we have termed (1) the biomedical frame, (2) the nurturing care frame and (3) the socioeconomic frame. Each frame corresponds to a common problem definition, proposed solutions and primary audience (individual, family, community). The dominant frames associated with each document are listed in Table 1, and Fig. 2 reveals the overlapping nature of sectoral alignments of the frames.

The biomedical frame refers to the location of FTD within the boundaries of maternal and child health, exemplified in the FTD rapid situational analysis report [46] and National Department of Health policies on maternal and child health [43]. The situational analysis and accompanying intervention framework [46] were developed by the perinatal task team, a technical support group in the provincial health department. The perinatal task team had examined resource allocation for neonatal health and, once the FTD got attention, extended the analysis to include the FTD, resulting in a document that was predominantly focused on biomedical interventions. In this frame, all behaviours or conditions in the FTD are expressed in terms of health and illness, and the problem to be addressed is defined principally as one of preventing maternal and child deaths.

This frame is also associated with the positioning of the health sector as having primary responsibility for propelling action on the FTD. Documents consistently mention that the health sector “*could, and should, take the lead in mobilising other government departments to address these broader social determinants of child health*” [47]. This is because the health sector interacts with pregnant women and children during the FTD and is thus the sector best placed to develop programmes and address the central problem of maternal and child mortality. Based on this problem definition, policy solutions lie in specific clinical-based strategies that emphasise clinical governance systems, including clinical guidelines, the training of health workers and health service improvement, while leaving out both the concept of nurturing care and socioeconomic factors that contribute to poor maternal and child health. These interventions therefore advocate for solutions that target individual patients through traditional health services.

The second frame outlined in Table 3 is the nurturing care frame, which is linked to two main ideas expressed in NIECD [9]. The first is the growing realisation that there is a need to complement or transform traditional maternal and child services into a more comprehensive approach that addresses existing gaps such as mental health and parental support. The second is the documented experiences of the lack of co-ordination resulting in fragmented early childhood development services between government agencies.

The articulated problem within this frame is one of a lack of support for childhood development that highlights issues surrounding the narrow range and poor quality of services offered to children. Policy programmes suggested to address this problem are therefore focused on stretching health services to include components of ‘nurturing care’, an idea presented by the 2016 *Lancet* series [14] and the 2018 Nurturing Care Framework for Early Childhood Development [48]. This frame appears in policies linked to the Social Development sector and plans released by the FTD executive committee. Although policy programmes addressing this second problem have a more holistic focus on child development, they are still largely grounded within the health sector and advocate for solutions that assimilate ideas from other sectors such as parenting support programmes or a focus on early stimulation. Policy solutions within this frame focus on the family level, by advocating for responsive caregiving and supporting caregivers to provide a nurturing environment for early childhood. An example is where community health worker programmes are encouraged to add a range of early childhood development services such as support to caregivers and home visits to the list of maternal health services they already provide:

“*Introduce a number of new services as an essential component of the comprehensive early childhood development programme to fill gaps identified in the range of services available, including: Early childhood development services provided through home visits by community health workers (CHWs) from conception until the child reaches the age of 2 years to vulnerable pregnant women, and post-natal services for women and children at high risk of poor early childhood development*” NIECD ([9], p. 55).

In contrast to the previous two frames, the community/societal frame links the FTD with the need to focus on the social determinants of health. Documents that explicitly link the FTD with this frame are subnational provincial strategies [13] and the FTD plan [49] released by the FTD executive committee. The shift from a focus on illness to addressing social determinants that affect health is a core strategy of the Province and the FTD is located within this narrative as an essential period to address vulnerabilities in early childhood. Although the impact of social determinants of health is well documented [2, 4], the concept of early life influences and child care starting at conception has also served to refocus attention on social disparities and health inequalities, as expressed in documents such as the Provincial Strategic Plan [13] and the *Lancet* series at a global level [50].

This frame promotes a sense of broader societal or political responsibility and stresses the involvement of the community in tandem with government sectors in addressing social determinants of health. Increased investment in the FTD and a strong focus on whole-of-society approaches, which involves engaging all aspects of society including citizens, civil society and the state, are thus advocated as an approach to navigate these challenges. While focusing on social issues contributing to poor health, proponents of this approach tend to present a general view of policy programmes, which broadens the options for interventions that impact communities as opposed to specific interventions that might target individuals. An example includes the following statement from the FTD plan in 2017: “*Begin to address social determinants of health relevant to the 1*^*st*^ *1000 Days*” ([51], p. 6) and the Cape Town Declaration of Wellness that proposes the following: “*Effective early childhood development is required to reduce vulnerabilities during childhood, adolescence and adulthood*” ([44], p. 2).

Based on the policy problems and solutions outlined above, the three frames overlap and can be linked to sectors as shown in Fig. 2.

### The missing ‘how’ within intersectoral collaboration discourse

The relevance of intersectoral collaboration for addressing FTD was acknowledged in many of the documents reviewed, showing an underlying acceptance of the idea. The importance of intersectoral action was framed as being crucial to achieving the SDGs and justified by previous experiences of poor child outcomes as a result of vertical services. However, the need for an intersectoral approach often appeared as a general statement, either at the beginning of the document, when the rationale of the FTD was laid out, or as a conclusion at the end of a document, with very little engagement on how collaboration was expected to unfold. A common example of this statement was as follows: “*The nature of the First 1000 Days also calls for intersectoral collaboration*” Situational Analysis ([46], p. 18).

Although not elaborated in detail, the documents did provide insights into what was considered to be intersectoral collaboration, which varied across documents to include referrals between professionals within the child space, government sectors working with non-governmental organisations and inter-departmental engagements within one sector. Additionally, health sector-specific documents would refer to intersectoral collaboration under the blanket umbrella of health promotion with no specific ideas of how this should be undertaken or what health promotion really involved. Common statements of “*Improve Maternal and Child wellbeing by initiating inter-sectoral health promotion programmes*” [46] or “*Intersectoral health promotion programmes should impact on children’s wellbeing*” [52] were included in the list of health-specific interventions that were proposed.

Among the few documents that engaged with governance arrangements appropriate to address the FTD, there was a dominant idea that inter-ministerial or multisector committees were the best way to ensure action across sectors [9, 50]. Subnational documents such as the Provincial Strategic Plan outlined the five provincial goals, governed through a provincial transversal management system, which provided the platform for the health sector to engage with others. Each provincial goal was managed by a steering committee that would address a range of projects to improve social determinants of health such as the FTD and include all the relevant sectors as part of the project.

## Discussion

This analysis reveals the influence of global ideas such as nurturing care on the agenda-setting process of the FTD in local contexts. Underpinning the literature on the FTD is the understanding that services for children will have to be planned and delivered in new ways that cross sectoral boundaries [14]. However, due to the scarcity of evidence regarding how intersectoral action unfolds, there is limited understanding about the best way to initiate and sustain collaboration across these sectoral boundaries [53]. Our analysis suggests that, even for initiatives that have widespread attention and support, such as the FTD, intersectoral collaboration cannot be taken as a given and faces a number of key obstacles.

First, policy solutions and programmes advocated during policy formulation often rely on the assumption that the problem is a given, the objectives are clear and that the policy solution provides the guide to achieving the objectives. However, many policy scholars have shown that problems and objectives are rarely pre-established and that framing of the problem determines the solution [28, 36]. Therefore, in policy spaces like the FTD, where multiple problem definitions exist, controversies will emerge if varied frames compete to define the problem [36]. Political contestation in this case becomes a struggle between different systems of meaning.

The difficulty of defining the ‘problem’ both within the FTD and when trying to address social determinants of health has been previously described [22, 54, 55]. Some of the reasons are linked to the uncertainty regarding the boundaries of the field and selecting priority interventions [22, 54, 55]. In the case of the FTD, the three problem definitions and solutions are largely influenced by the health and social development sectors. The limited focus on safety-related interventions and early stimulation point to the need for further engagement with sectors involved in education and safety. The three frames are not entirely incompatible and all contribute to achieving the FTD; they are similar in two ways. The first is that there is agreement of the need to prioritise action for maternal and child health within the 2-year period. The second is the role the health sector has to play in acting as a leader or driver of action for the FTD. However, the point of contention becomes the level of involvement of other sectors.

The lack of consensus regarding the roles of various sectors impacts ownership of the initiative, creating uncertainty regarding which governance or funding structures should be established and this could result in policy stasis of initiatives such as the FTD. Each of the three frames propose policy responses that have an impact in motivating intersectoral action as they form the basis for decisions regarding resources and governance arrangements. Frames also determine the legitimacy of actors who can participate in policy formulation processes. The biomedical frame, for example, proposes solutions that require knowledge of the health sector and clinical governance and leaves little opportunity for cross-sector engagements. The nurturing care frame allows for intersectoral work in response to particular initiatives or opportunities, while the socioeconomic frame proposes an integrated, whole-of-society approach. The package of ideas within these frames either support or hinder the potential for collaborative working.

Additionally, this study reveals that policies governing the delivery of child services rely on the assumption that intersectoral action is important and will take place willingly, but leaves the question of how unanswered. Effective collaboration does not happen effortlessly; it requires a deliberate process with the alignment of a range of factors, including favourable initial starting conditions of partnerships, leadership and governance, and capacity, amongst others [53, 56–58].

At the core of intersectoral collaboration and an essential starting point is having an initial agreement on problem definitions [56, 57, 59]. If treated as equal, the three frames for the FTD can be complementary but require negotiation between key sectors and organisations involved to enable agreement on adequate starting points, interventions and governance arrangements. Ansel and Gash refer to a range of terms such as a shared understanding, common mission and shared vision, all implying that collaborative partners jointly articulate what they can achieve together [57]. Underpinning the development of shared meanings and articulation of a common purpose is the mutual understanding of each other’s interests and positions [58].

Collaborative governance theories approach the question of divergent interests by suggesting a principled engagement process where stakeholders discover and deliberate between common and varied interests and eventually arrive at joint determinations or definitions of goals. The principled engagement process entails collaborative learning, acknowledging and expecting conflict, ongoing communication and trust [58]. Time is required to reflect on differing points of view and develop shared understanding [60]. The ability to manage conflicts is also crucial in collaborative engagements as existing differences can be worsened by power differentials or competition [61]. Additionally, fostering collaborative learning implies that collaborative engagements need to be flexible enough to allow reframing in order to advance the achievement of goals. Framing can also be approached deliberately to define problems in ways that appeal to the interests of other actors and can be negotiated over time as partners move towards achieving a common goal. Framing the problem in a way that actors can identify with the common goals is useful for stakeholders outside the health sector who have limited knowledge regarding the problem or solutions and who may be unsure of the benefits of collaboration [48].

Failure to view collaboration as a continuous learning and relationship-building process, where frames are surfaced and negotiated through an ongoing communicative process, may lead to the dominance of one problem definition over others as initiatives move towards implementation. Which problem definition/s will dominate depends on a range of factors, which include power and resources of claimants, how the issue is portrayed, venues where problems are debated, who claims ownership for the problem, availability of policy solutions for the problem, and the fit between problem definition and context [28, 36].

Previous studies [39, 40] that have examined intersectoral action on social determinants of health have shown the dominance of the biomedical and behavioural perspectives on health and illness. This has been associated with an individualised approach to health, which focuses on promoting the change of individual behaviour. Biomedical perspectives can hinder the consideration of other social, political and structural options that are necessary to address the issue [40]. In cases where the health sector leads the intersectoral initiative, there is a risk that biomedical framings are most likely to win. One reason could be that such framings tend towards specific technical solutions, whereas addressing social determinants of health may require less specific policy options over a long period of time and flexibility to various approaches in different settings. This lack of specificity of solutions can seem less desirable when compared to biomedical interventions that can link cause and effects and may have readily visible outcomes.

## Conclusion

Our study offers insight into the role of frames during early decision-making processes of intersectoral collaboration. The challenges for initiating intersectoral action revealed in the study should be considered during the formulation process of other similar initiatives. The first is the insufficient attention in policies on how intersectoral action should unfold. Secondly, the lack of co-ordination and alignment across policies of relevant sectors. Thirdly, not explicitly allowing for negotiation of differing frames of the problem and solutions. Collaborative governance literature points to the crucial role of fostering collaborative learning and open communication through a principled engagement process that allows different frames to be surfaced and negotiated, and the formulation of common goals over time. This process of discovery and deliberation of varied interests should be considered a vital starting point of collaborative engagements, which is relevant for the FTD process as it unfolds in the Provincial context and for other settings embarking on addressing ECD-related policy action. As a limitation to this study, we acknowledge that the results of this paper are not necessarily generalisable to all problems requiring intersectoral collaboration, and in all other contexts. However, this paper demonstrates that differing frames might be a significant issue in complex multisector policy initiatives with multiple possible solutions; this is typically the case in multisector initiatives addressing stages in the life course (e.g. childhood, adolescence, old age).

## Supplementary information

**Additional file 1.** Document Extraction Sheet. The document displays the extraction process of all the documents that were analysed.

## Data Availability

All data generated or analysed during this study are included in this published article as Additional file 1.

## Abbreviations

ECD: Early Childhood Development
FTD: First 1000 Days
MDGs: Millennium Development Goals
NIECD: National Integrated Early Childhood Development
SDGs: Sustainable Development Goals
